# An epidemiological investigation of gallstone disease among patients admitted to Shahid Rahimi teaching hospital in Khorramabad in 2016-2020

**DOI:** 10.4314/ahs.v23i2.50

**Published:** 2023-06

**Authors:** Saleh Azadbakht, Raziyeh Parvaee, Samad Darabian

**Affiliations:** 1 Department of Internal Medicine, School of Medicine, Lorestan University of Medical Sciences, Khorramabad, Iran; 2 Student Research Committee, School of Medicine, Lorestan University of Medical Sciences, Khorramabad, Iran; 3 Department of Statistics and Epidemiology, School of Health, Lorestan University of Medical Sciences, Khorramabad, Iran

**Keywords:** Gallstones, epidemiology, risk factors, Khorramabad

## Abstract

**Background:**

Gallstones are a significant global health issue, imposing enormous costs to patients and the healthcare system, annually.

**Objectives:**

This epidemiological study aimed to explore the prevalence of gallstones among inpatients who were admitted to Shahid Rahimi Hospital in Khorramabad City, Iran, from 2016 to 2020.

**Methods:**

This was a retrospective and descriptive-analytical study. The study population included patients who were admitted to Shahid Rahimi Hospital in Khorramabad City, Iran, who underwent abdominal ultrasounds from 2016 to 2020. The required data were collected using a checklist and patients' records. The logistic regression analysis method was used to analyse the obtained data in SPSS at the significance level of P<0.05.

**Results:**

Of the 927 explored subjects, 232(25%) presented gallstones. The mean age of the patients with gallstones was 62.9 years. The most frequent symptom in individuals with gallstones was right upper quadriceps abdominal pain (63%). There was a significant relationship between gallstone and age, gender, epigastric pain, upper quadrant pain, hypertension, anemia, and family history.

**Conclusion:**

The present study findings indicated that increasing age, female gender, the lack of epigastric pain, upper quadrant pain, anemia, hypertension, and a family history of this disease increased the risk of generating gallstone disease.

## Introduction

Gallstones are a significant health concern in developed societies^1^. This disease is among the major reasons for hospitalization due to gastrointestinal diseases in European countries^2^. Gallstones are mainly composed of cholesterol, bilirubin, and calcium salts, as well as slight amounts of protein and other substances^3^,^4^. Western countries categorize gallstones into two groups, as follows: 1. Cholesterol gallstones, containing >50% cholesterol and are responsible for approximately 75%-80% of gallstone cases. 2- Pigment gallstones, with <30% of their weight belonging to cholesterol^5^. Gallstone Disease (GSD) imposes high costs to the healthcare system; the direct and indirect financial burden of GSD accounts for 6.2 billion pounds in the United States per year, i.e., a major health concern^6^-^8^.

With an estimated 1.8 million outpatient visits per year, GSD is among the main reasons for admission to hospitals concerning gastrointestinal conditions 9. The prevalence of gallstones varies from 0.1% to 50.5% worldwide^10^. The prevalence of gallstones is on the rise in Europe and the USA^11^. In the USA, according to the Third National Health and Nutrition Survey (NHANES-III), the overall prevalence of gallstones was measured to be 7.9% and 16.6% in males and females, in sequence^12^. In Iran, the prevalence of GSD in Amol, Birjand, and southern Iran was approximately calculated as 0.8%, 2.5%, and 4.7%, respectively^13^.

Epidemiological studies outlined an association between GSD and a higher risk of ischemic heart disease and overall mortality and disease-induced mortality (cardiovascular disease & cancer). These correlations were statistically confirmed after adjusting potential disruptors, including traditional risk factors (e.g., body weight, smoking, physical activity, diabetes, hypertension, & hypercholesterolemia)^14^-^16^. Numerous individuals with gallstones present no significant symptoms. Additionally, about 25%-50% of these patients, experience complications that require cholecystectomy. Acute cholecystitis is the most common complication of gallstones induced by the dilatation and inflammation of the gallbladder following obstruction caused by stones in the cystic duct. Secondary bacterial infection also occurs in 50% of all cases; accordingly, multiple symptoms may be manifested in this regard^17^.

From a public health perspective, not only it is essential to explore the epidemiology of GSD regionally, but it is also possible to examine the demographic and biological markers associated with the development of GSD. Therefore, considering the importance, frequency, and significant costs that patients with gallstones and the health system incur, it seems necessary to study the epidemiology, signs, and symptoms of GSD. Therefore, this epidemiological study investigated the prevalence of gallstones in patients who were admitted to Shahid Rahimi Hospital in Khorramabad City, Iran, from 2016 to 2020.

## Methods

This retrospective and descriptive-analytical study was performed to investigate the epidemiology of GSD in patients who were admitted to Shahid Rahimi Hospital in Khorramabad City, Iran, in 2016-2020. The study sample was selected using a systematic random sampling method. The study population included all hospitalized patients present with gallbladder and bile duct ultrasounds. In total, 927 records of all patients who were admitted to the study Hospital and underwent abdominal ultrasound were collected. Next, the necessary information, such as the presence of gallstones, age, gender, as well as clinical symptoms, including epigastric pain, right upper quadrant pain, recent weight loss, underlying diseases, such as hypertension and diabetes, anemia, and a family history of gallstones were extracted from the patients' records. These data were collected by a researcher-designed checklist.

The obtained data were analysed in SPSS. Frequency and ratio values for categorized variables and mean (SD) for continuous variables were used to describe the characteristics of the study subjects. Logistic regression analysis was employed to investigate the relationship between independent variables and GSD. The significance level of the test was considered P<0.05.

## Results

Of the total 927 explored cases, 232(25%) individuals presented GSD. The mean±SD age of the study subjects was 57.52±19.71 years. Moreover, 441(47.6%) patients were male. Furthermore, 558(60.2%) subjects lived in urban areas. Besides, 252(27.2%) patients experienced epigastric pain; 202(21.8%) reported right upper quadrant pain; 286(30.9%) subjects encountered hypertension; 156(16.8%) had diabetes, and 320(34.5%) patients manifested anemia. Additionally, 177(19.1%) subjects had lost >10 kg in the last 6 months. Also, 196(21.1%) subjects reported a family history of GSD.

Logistic regression analysis was used to test the fit of the data. The Odds Ratio (OR) was obtained for age (P=0.00, OR=1.017, 95%CI: 1.011-1.021). Thus, there was a significant relationship between age and GSD; accordingly, per unit increase in age, the odds of developing GSD were elevated by 1.7%. The OR was also measured for gender (P=0.00, OR=0.44, 95%CI: 0.29-0.66). As a result, a significant relationship was observed between gender and GSD; the odds of developing GSD were 0.44% lower in men and women. The OR was calculated for epigastric pain (P=0.00, OR=0.45, 95%CI: 0.28-0.72). There existed a significant relationship between epigastric pain and GSD. Moreover, the chances of developing the disease were 0.45% less in those with epigastric pain, compared to the subjects without epigastric pain. The OR was computed for right upper quadrant pain (P=0.00, OR=25.62, 95%CI: 16.34-40.17). The obtained data indicated that those with upper quadrant pain were 25.6 times more prone to encounter GSD. The OR was calculated for anemia (P=0.00, OR=1.95, 95%CI: 1.30-2.94).

The collected results outlined a relationship between anemia and GSD, in which the prevalence of gallstones in those with anemia was almost twice higher than those without anemia. The OR was measured for blood pressure levels (P=0.01, OR=1.84, 95%CI: 1.15-2.95). Thus, there was an association between blood pressure levels and the bile duct. In other words, the prevalence of GSD in those with hypertension was 1.84 times higher than that in patients without such a condition. The OR was computed for family history (P=0.00, OR=2.98, 95%CI: 1.95-4.57). Accordingly, a relationship was detected between family history and GSD, with the prevalence of GSD being 3 times higher in those with a GSD family history, compared to the participants without such a history. There was no significant correlation between residence place (P=.159, OR=1.33, 95%CI: 0.89-2.02), diabetes (P=0.88, OR=1.03, 95%CI: 0.61-1.72), and weight loss over the past 6 months (P=0.71, OR=1.10, 95%CI: 0.65-1.84), as well as GSD (Table 2).

**Table 2:** The logistic regression analysis data on the risk factors associated with GSD (N=924)

Characteristic		β	SD	OR	95%CI	P
Age, y		0.017	0.006	1.017	1.011-1.021	0.00

Gender	Male	-0.820	0.208	0.44	0.29-0.66	0.00
	Female	Comparator				

Place of residence	Rural	0.292	0.207	1.33	0.89-2.02	0.159
	Urban	Comparator				

Epigastric pain	Yes	-0.799	0.241	0.45	0.28-0.72	0.00
	No	Comparator				

Right upper quadrant pain	Yes	3.244	0.229	25.62	16.34-40.17	0.00
	No	Comparator				

Hypertension	Yes	0.611	0.241	1.84	1.15-2.95	0.01
	No	Comparator				

Diabetes	Yes	0.033	0.262	1.03	0.61-1.72	0.88
	No	Comparator				

Anemia	Yes	0.672	0.208	1.95	1.30-2.94	0.01
	No	Comparator				

Wight loss	>10kg/6m	0.095	0.264	1.10	0.65-1.84	0.71
	<10kg/6m	Comparator				

Family history	Yes	1.094	0.218	2.98	1.95-4.57	0.00
	No	Comparator				

## Discussion

Of the examined 927 cases, 232(25%) presented GSD, and 695(75%) subjects were healthy. Thus, the prevalence of GSD was estimated to be 25%. The prevalence rate obtained in the present research was almost consistent with those of the previous studies^20^-^20^. However, some investigations, like the study by Ansari-Moghaddam et al. reported the general prevalence of GSD as 2.4% in the southeast of Iran^21^.

Zamani et al. (2014) assessed the prevalence of gallstones in asymptomatic patients in Amol to be 0.8%, i.e., much lower than that suggested by other domestic studies. Such data discrepancy could be attributed to the nature of their study, i.e., restricted to asymptomatic patients or those fostering a healthier diet and lifestyle^22^. In the study by Jun et al. in China, the prevalence of GSD was measured as 5.7%^23^. In the study by Chen et al. (2006) in Taiwan, the general prevalence of GSD was calculated to be 5% 24. Such data indicated a lower prevalence of GSD in Eastern countries, which can be due to genetics and lifestyle differences^3^, ^25^.

These variations in prevalence rates may be largely attributed to dietary differences, such as higher calorie, refined sugars, and saturated fats intake, a less sedentary lifestyle, and reduced fiber intake, as well as genetic characteristics^20^. The mean age of individuals with GSD was 62.9 years. Additionally, the logistic regression analysis results determined a significant relationship between age and GSD, as the prevalence of gallstones increases with age. The present study findings were consistent with those of the previous studies^16^, ^21^, ^22^, ^26^-^28^.

This could be because the underlying conditions associated with GSD, such as hypertension and diabetes, and high blood cholesterol levels are more frequent at older ages. The collected data addressed a significant relationship between gender and GSD; the frequency of GSD was lower in males than females. These results were in line with those of similar studies^16^, ^20^, ^23^, ^29^. The high prevalence of GSD in women might concern female sex hormones. The use of birth control pills or estrogen therapy after menopause are significant risk factors for the formation of cholesterol gallstones^30^.

Estrogen increases cholesterol secretion and decreases bile salt secretion, while progestins decline bile salt secretion and disrupt gallbladder emptying, leading to stasis^31^. The current research results suggested no significant relationship between GSD and the place of residence. In other words, living in urban or rural areas does not play an effective role in increasing the incidence of generating GSD. There is no consensus in this respect in the literature. For example, Yang et al. compared participants with and without gallstones and concluded that the subjects with GSD mostly lived in cities^23^.

However, Zamani et al. performed an investigation in northern Iran among an asymptomatic population. The relevant results highlighted that the risk of developing GSD was associated with rural life^22^. Such data discrepancy can probably be explained by differences in lifestyle, diet, and genetic characteristics in the urban and rural residents of various regions. We observed a significant relationship between right upper quadrant abdominal pain and GSD; thus, the prevalence of GSD in those with right upper quadrant pain was 25.6 times higher than the individuals without such conditions. Vahed et al. argued that the most frequent pain complaint was related to the upper right quadrant, accounting for 95.7% of the cases^26^.

In Berger et al.'s study, the most prevalent complaint in males respected right upper quadrant pain in the abdomen^32^. As per Froutan et al. and Nimanya et al., the most common symptom in patients with GSM was abdominal pain (69.7%) (18, 19). The achieved results signified a correlation between experience epigastric pain and GSM; the prevalence of gallstones in those who manifested epigastric pain was 45% less than their counterparts without epigastric pain. These data were inconsistent with those of Nimanya et al., where a majority of patients reported epigastric pain^19^. Such data differences concerning epigastric pain may be because the prevalence of dyspepsia and peptic ulcer might have been higher; consequently, non-gallstone epigastric pain was more frequent in the study population. Further studies are recommended in this area. Chavez et al. and Chen et al. also supported an association between blood pressure and gallstones stated by Weikert and colleagues^28^, ^33^, ^34^.

This finding was consistent with those of our study; the prevalence of gallstones in those with hypertension was 1.8 times higher than in the subjects without hypertension. The underlying mechanism behind the impact of hypertension and the enhanced risk of GSD remains undiscovered. Some researchers have suggested that this association may be explained by the function of insulin in hypertension^34^. The current research data demonstrated no significant relationship between diabetes and gallstones. These results were inconsistent with those of numerous similar studies^18^, ^23^, ^27^, ^34^. It remains unclear how diabetes elevates the risk of gallstones. However, hypertriglyceridemia, autonomic neuropathy (leading to gallbladder immobility & biliary stasis), and hyperinsulinemia were suggested as characteristics affecting the increased risk of gallstones in patients with diabetes^35^.

This data discrepancy may be possibly attributed to the level of awareness of diabetes status between the sample of the current research and other investigations. This is because the required information was extracted from the patient's self-reported records; thus, the medical history provided by the patients might be incomplete. As a result, it is suggested that further studies be performed to also monitor patients' blood glucose levels. We detected a significant correlation between anemia and gallstones. Accordingly, the prevalence of GSD in individuals with anemia was almost twice as high as the non-GSD cases. This finding was in line with those of some studies^28^,^33^,^34^,^36^.

Iron deficiency leads to anemia and plays an essential role in excessive bile saturation, leading to the formation of stones in the gallbladder. Iron acts as a coenzyme for the enzyme nitric oxide synthetase and produces nitric oxide to maintain gallbladder tone and natural relaxation. Changes in the movement of the gallbladder and sphincter of Oddi lead to biliary stasis in the generation of cholesterol crystals, i.e., reported with iron deficiency (36). We detected no significant relationship between a >10kg weight loss in the last 6 months and developing gallstones^19^, ^37^, ^38^.

These results were inconsistent with those of relevant studies. Such data discrepancy can be explained as follows: in the present study, a weight loss of more than 10 kg was explored in 6 months, in which the pattern and rate of this type of weight loss differ from rapid weight loss due to following highly restrictive calorie diets. A weight loss of more than 1.5 kg per week following obesity surgery increases the risk of gallstones formation^1^,^39^. Hemminki et al., in Sweden, Jessri et al. in Iran, and Hsing et al., in Shanghai revealed that family history is associated with an increased risk of developing gallstones^37^,^40^,^41^. These studies were consistent with those of our study, suggesting a significant relationship between family history and generating gallstones. In other words, the prevalence of gallstones in those with a family history is approximately 3 times higher than that in individuals without such a history.

## Conclusion

The present study data revealed that higher age, female gender, the lack of epigastric pain, upper quadrant pain, anemia, hypertension, and a family history of this disease increase the risk of developing GSD.

## Figures and Tables

**Table 1:** The demographic characteristics of the study participants (N=924)

Characteristic		GSD		Total Mean (SD)
		Yes Mean (SD)	No Mean (SD)	
Age, y		62.91(18.92)	55.72(19.65)	57.52(19.71)

Gender	Male	36.2(84)	357(51.4)	441(47.6)
	Female	148(63.8)	338(48.6)	486(52.4)

Place of residence	Rural	145(62.5)	413(59.4)	558(60.2)
	Urban	87(37.5)	282(40.6)	369(39.8)

Epigastric pain	Yes	55(23.7)	197(28.3)	252(27.2)
	No	177(76.3)	498(71.7)	675(72.8)

Right upper quadrant pain	Yes	146(62.9)	56(8.1)	202(21.8)
	No	86(37.1)	639(91.9)	725(78.2)

Hypertension	Yes	88(37.9)	198(28.5)	286(30.9)
	No	144(62.1)	497(71.5)	641(69.1)

Diabetes	Yes	53(22.8)	103(14.8)	156(16.8)
	No	179(77.2)	592(85.2)	771(83.2)

Anemia	Yes	99(42.7)	221(31.8)	320(34.5)
	No	133(57.3)	474(68.2)	607(65.5)

Wight loss	>10kg/6m	48(20.7)	129(18.6)	177(19.1)
	<10kg/6m	184(79.3)	566(81.4)	750(80.9)

Family history	Yes	92(39.7)	104(15)	196(21.1)
	No	140(60.3)	591(85)	731(78.9)
